# Homology Characteristics of EEG and EMG for Lower Limb Voluntary Movement Intention

**DOI:** 10.3389/fnbot.2021.642607

**Published:** 2021-06-18

**Authors:** Xiaodong Zhang, Hanzhe Li, Zhufeng Lu, Gui Yin

**Affiliations:** ^1^School of Mechanical Engineering, Xi'an Jiaotong University, Xi'an, China; ^2^Shaanxi Key Laboratory of Intelligent Robots, Xi'an Jiaotong University, Xi'an, China

**Keywords:** numerical simulation, homology analysis, EEG, EMG, coherence

## Abstract

In the field of lower limb exoskeletons, besides its electromechanical system design and control, attention has been paid to realizing the linkage of exoskeleton robots to humans via electroencephalography (EEG) and electromyography (EMG). However, even the state of the art performance of lower limb voluntary movement intention decoding still faces many obstacles. In the following work, focusing on the perspective of the inner mechanism, a homology characteristic of EEG and EMG for lower limb voluntary movement intention was conducted. A mathematical model of EEG and EMG was built based on its mechanism, which consists of a neural mass model (NMM), neuromuscular junction model, EMG generation model, decoding model, and musculoskeletal biomechanical model. The mechanism analysis and simulation results demonstrated that EEG and EMG signals were both excited by the same movement intention with a response time difference. To assess the efficiency of the proposed model, a synchronous acquisition system for EEG and EMG was constructed to analyze the homology and response time difference from EEG and EMG signals in the limb movement intention. An effective method of wavelet coherence was used to analyze the internal correlation between EEG and EMG signals in the same limb movement intention. To further prove the effectiveness of the hypothesis in this paper, six subjects were involved in the experiments. The experimental results demonstrated that there was a strong EEG-EMG coherence at 1 Hz around movement onset, and the phase of EEG was leading the EMG. Both the simulation and experimental results revealed that EEG and EMG are homologous, and the response time of the EEG signals are earlier than EMG signals during the limb movement intention. This work can provide a theoretical basis for the feasibility of EEG-based pre-perception and fusion perception of EEG and EMG in human movement detection.

## Introduction

With the intensifying of the aging problem, and with the military and civilian goals to amplify the human ability, many studies have focused on the development of robotics to break through human motor limitations such as terrain conditions or individual ability (Al-Quraishi et al., 2018). Lower limb exoskeleton robotics is one of the eminent research areas to develop load augmentation, which could further enhance the lower limb's ability, reduce the energy consumption of humans, and increase a human's load capacity (Qiuzhi et al., 2016). Recent notable research on exoskeleton robotics as well as its related technology have extended applications to practical areas. Besides the assistive tool for healthy people, lower limb exoskeleton robotics is also expected to broadly meet social requirements in the fields of medical treatment, walking-assistance for senior and disabled people, industrial and agricultural production, and other fields (Rupal et al., 2017). Despite this field having attracted a considerable level of attention over the last few years, there are still some problems with the prediction of human lower limb voluntary movement intention (Chen et al., 2016).

Nowadays, the mainstream way for human movement intention detection is mainly based on bioelectrical signals or force and position information. For bioelectrical signals, electroencephalogram (EEG) and electromyography (EMG) are especially favored in further analyzing the human movement intention. As for the force and position information methods, since it can only be obtained after limb movement, its shortage in time delay is inevitable. Furthermore, the cost time of information processing and electromechanical system response further reduce real-time performance (Yi et al., 2015). Hence, several efforts have been made to apply EEG and EMG for robotic control, since it can solve this problem very well.

Due to the advantages of EEG and EMG signals, those signals are used to decode the different human movement information. Thirty-two EEG features from 64-channel EEG signals were used to decode multiple classes of different upper limb movements, the average accuracy was above 90%, this is due to the optimal feature set combination which would yield a relatively high decoding accuracy (Samuel et al., 2017). EMG signals contain motor/neural information from which limb movement intent could be identified, and it can recognize more kinds of movement patterns (Samuel et al., 2019). But with the limitation of current technologies, the decoding performance is not very satisfactory under certain conditions. For example, when only using EEG, it is difficult to achieve a satisfactory accuracy, and when only using EMG it is hard to guarantee the stability of recognition. Rui et al. classified the facial action to control a prosthesis, and the results showed that the performance of EMG-based control is better than EEG-based control (Rui et al., 2018b; Xiaodong et al., 2020). Therefore, the fusion method of EEG and EMG signals emerge as the times require, it can improve decoding performance and stability (Tejedor et al., 2019). Leeb et al. discussed the decision fusion of EEG and EMG, and the results showed that different proportion of EMG and EEG fusion had a better and more stable performance (Leeb et al., 2011). Li et al. applied EEG and EMG electrode arrangements using the Sequential Forward Selection (SFS) algorithm, this study demonstrated the feasibility of fusing EMG and EEG signals toward improving motion classification accuracy (Xiangxin et al., 2017). Hazarika presented the real-time implementation of a feature fusion-based learning algorithm using multi-domain discriminant correlation analysis (MDCA), the results demonstrated the effectiveness and reliability of the fusion of EEG and EMG (Hazarika et al., 2019).

However, there are some deficiencies in the current methods of EEG and EEG fusion. Although information of EEG and EMG is fused, its contents are inconsistent. Such inconsistency results from the difference in the response time of EEG and EMG, which leads to the differences in the contents of EEG and EMG signals collected at the same time. Therefore, the performance improvement of EEG and EMG fusion based detection is limited. Only few researchers devoted to explore the mechanism of EEG and EMG for lower limb voluntary movement intention, which can clarify the internal correlation of EEG and EMG and provide a theoretical support for designing a fusion method of EEG and EMG. Hence, this paper presents a numerical simulation and homology analysis of EEG and EMG with lower limb voluntary movement intention. We hypothesized that EEG and EMG signals are homologous and the EEG signal response is earlier than the EMG signal response. In this paper, a mathematical model of EEG and EMG to simulate the EEG and EMG signals and analyze the homology is presented and validated. The organization of this work is as follows. Section Methodologies, including the brain cognitive model with multiple input-multiple output, the mechanism of EEG and EMG and analysis method for homology characteristics of EEG and EMG. Section Numerical Simulation of EEG and EMG for Lower Limb Voluntary Movement Intention. Section Experimental Verification for Homology Characteristic of EEG and EMG, which describes the simulation and experimental results as well as corresponding homology analysis. Sections Discussion and Conclusion, respectively.

## Methodologies

### Brain Cognitive Model With Multiple-Input Multiple-Output

**Figure 1:**
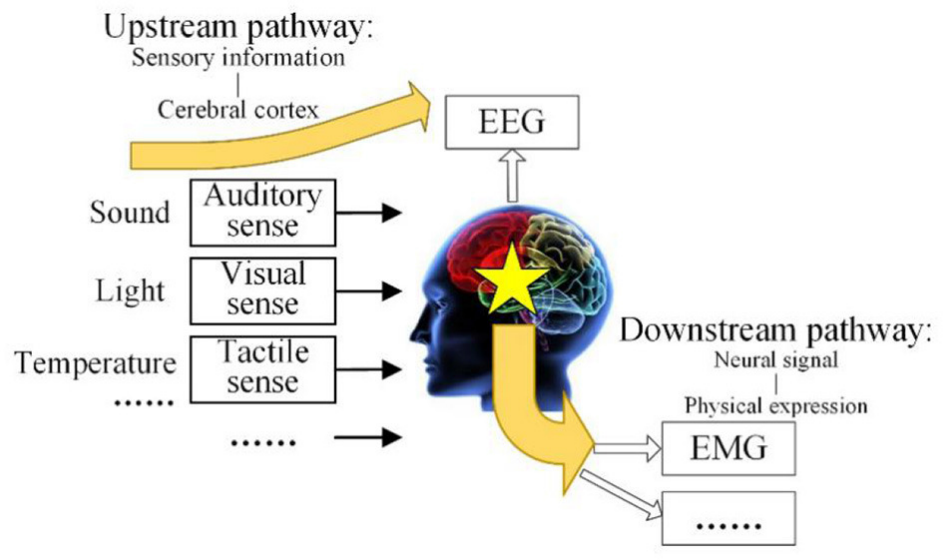
Brain cognitive model with multiple-input multiple-output.

The human brain is the center where people receive information, process it, and make decisions, so constant efforts are committed to explore and analyze the human brain to better serve human beings. Since Hans Berger recorded EEG signals for the first time in 1924, many scholars have carried out a lot of research work on the establishment of the brain model, EEG signals acquisition, EEG information decoding, and brain computer interfaces (Xiaodong et al., 2014). Therefore, the inevitable problem has arisen in brain control technology of how to establish a universal brain cognitive model, develop a corresponding neural control model, and effectively apply them to robotic interactive controls. In this paper, a brain cognitive model with multiple-input multiple-output was proposed, in which the brain receives outside information and generates a response so that the relative neural pathways are set up as shown in Figure 1 (Xiaodong, 2018).

The brain cognitive model with multiple-input multiple-output consists of an upstream pathway and downstream pathway. The upstream pathway is the revelation of the mechanism of EEG, from sensory information to the cerebral cortex. The upstream pathway shows that the environmental physical information comes from the human visual sense, auditory sense, tactile sense, and other senses, and it will be processed in the brain with the existing intelligent knowledge so that some brain response is excited. The EEG signals will be acquired in the cerebral cortex, where the brain response transmits upward. The downstream pathway is the revelation of the mechanism of EMG, from the brain response to the neural signal, and then to physical expression. The downstream pathway shows that the control signals from brain are transmitted to the limb through the nervous system, after that the external expression of EMG response and limb movement is generated. This model systematically expounds the source of EEG and EMG and reveals the internal relationship between them.

### Mechanism of EEG and EMG for Lower Limb Voluntary Movement Intention

**Figure 2:**
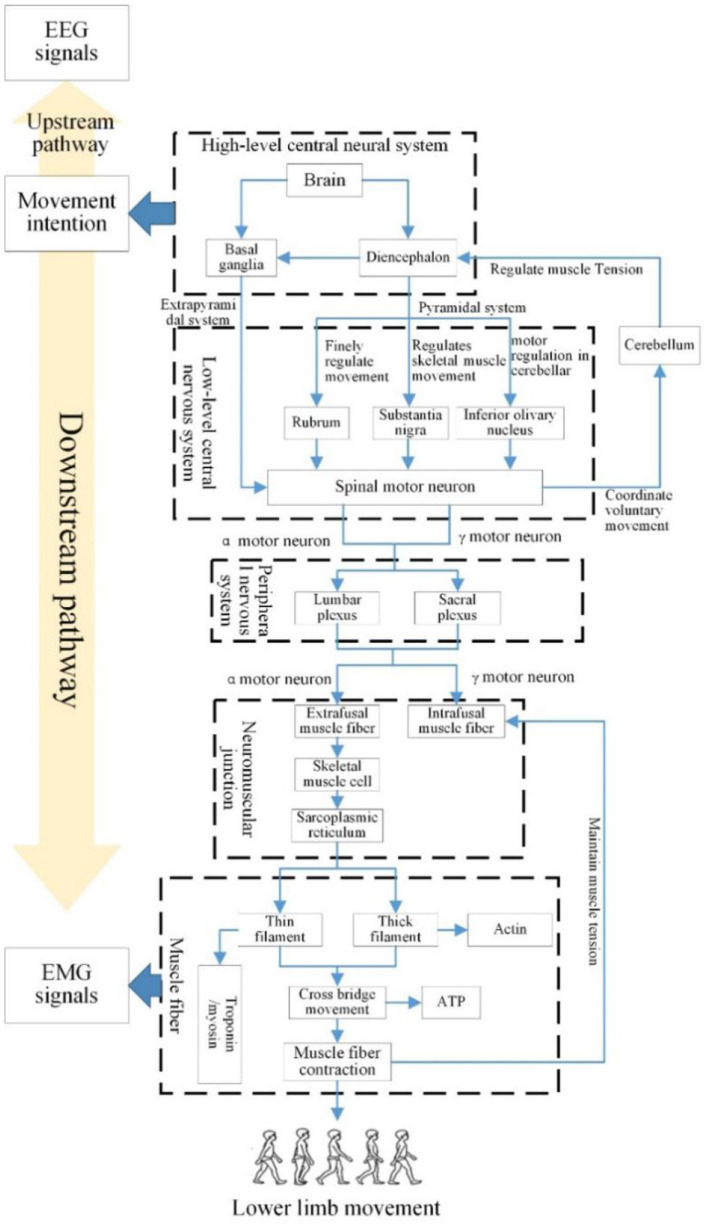
Neural pathway of lower limb voluntary movement.

On the basis of the proposed brain cognitive model, the mechanism of EEG and EMG of lower limb voluntary movement intention was refined. Human limb movement can be divided into evoked and voluntary movement. Evoked movement is caused by environmental stimulation, which is a response to meet the environmental needs. Voluntary movement is generated by the human's intention to achieve an intended purpose (Waszak et al., 2005; Herwig et al., 2007). The neural pathway of evoked and voluntary movement was shown in Figure 2.

In voluntary movement, the pre-motor cortex and anterior cingulate cortex play important roles in the evocation of movement (Mueller et al., 2007). Regarding evoked movement, the ventral/dorsal pre-motor cortex and posterior parietal cortex are crucial (Obhi and Haggard, 2004; Waszak et al., 2005). The information of evoked and voluntary movement is processed by pre-motor cortex. Processed information will be transmitted to limbs through the peripheral nervous system, causing limb movement. Neuron activity in the motor cortex generates an EEG response including a negative low-frequency potential, which is defined as readiness potential (RP). RP is an ERP (event-related potential), and also is important component of MRCP (movement-related cortical potentials) (Verleger, 2003), it is a remarkable spatiotemporal feature that can be used to analyze the homology and decode movement intention.

### Analysis Method for Homology Characteristics of EEG and EMG

The coherence analysis of EEG and EMG is an effective method for homology analysis between two signals representing brain and muscles, respectively. The wavelet coherence gives full play to transform characteristics in the time–frequency domain, which is an important method for processing nonstationary signals (Xugang et al., 2020). In this work, signal sequence *x* (*t*) and *y* (*t*) denotes EEG and EMG signals respectively. *W*_*x*_ (*a, b*) denotes the wavelet coefficients, it is obtained by convoluting the scaled wavelet function with *x* (*t*).

(1) $$\begin{array}{l} W_{x}\left(a,b\right)=\langle x,\psi_{a,b}\rangle =\frac{1}{\sqrt{a}}\int_{-\infty }^{\infty }x\left(t\right)\psi *\left(\frac{t-b}{a}\right)dt \end{array}$$

Where *a* is the wavelet scale, *b* is the smoothing parameter, ^*^ is the conjugate, ψ[ (*t*–*b*)/*a*] is the wavelet basis function.

|*W*_*xy*_ (*a, b*)| is the cross-wavelet trans-formation of *x* (*t*) and *y* (*t*). So, the wavelet cross-spectrum of the EEG signals *x* (*t*) and the EMG signals *y* (*t*) can be expressed as follows:

(2) $$\begin{array}{l} \left|W_{xy}\left(a,b\right)|=|W_{x}\left(a,b\right)W_{y}\left(a,b\right)\right| \end{array}$$

The wavelet spectrum needs to be smoothened to calculate the synchronization information of EEG and EMG signals, it is defined as follows:

(3) $$\begin{array}{l} S\left(W\right)=S_{a}\left[S_{t}\left(W\right)\right] \end{array}$$

Where *S*_*a*_ and *S*_*t*_ are the smoothing operation on the scale axis and time axis, respectively.

(4) $$\begin{array}{l} S_{a}\left(W\left(a,b\right)\right)=W\left(a,b\right)*c_{1}\Pi \left(0.6a\right)\\ S_{t}\left(W\left(a,b\right)\right)=W\left(a,b\right)*c_{2}^{-\frac{t^{2}}{2a^{2}}} \end{array}$$

Where, *c*_1_ and *c*_2_ are the normalization coefficients, and ∏ is a matrix function.

The absolute value of the normalized smooth cross-wavelet spectrum is wavelet coherence (Lachaux et al., 2002). Thus, the wavelet coherence coefficient of *x* (*t*) and *y* (t) is as follows:

(5) $$\begin{array}{l} Wco_{xy}\left(a,b\right)=\frac{\left|S\left(W_{xy}\left(a,b\right)\right)\right|}{\sqrt{S\left({\left|W_{x}\left(a,b\right)\right|}^{2}\right)S\left({\left|W_{y}\left(a,b\right)\right|}^{2}\right)}} \end{array}$$

The wavelet coherence coefficient is in the range of 0–1, the greater the value, the stronger the coherence.

## Numerical Simulation of EEG and EMG for Lower Limb Voluntary Movement Intention

### Mathematical Model of EEG and EMG

**Figure 3:**
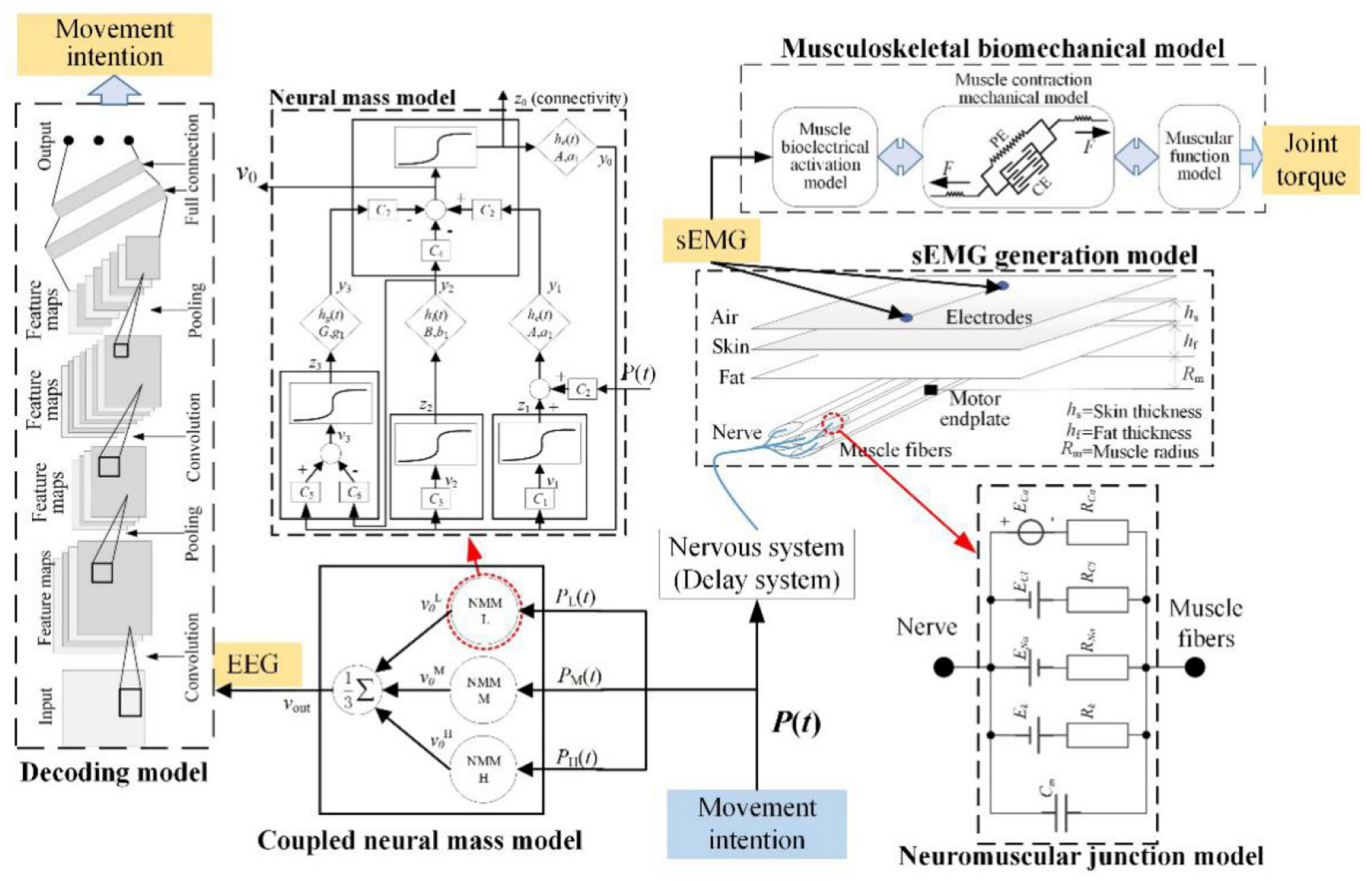
Mathematical model of EEG and EMG for movement intention.

The mechanisms of EEG and EMG signals during lower limb voluntary movement intention provide the theoretical basis for robot control system (Hanzhe et al., 2019b), it can be seen that when humans generate movement intention, it is first processed in the brain to generate EEG signals, and then transmitted to limbs by neural pathway, which cause limb movement to generate surface EMG (sEMG) signals through the peripheral nervous system. So, the mathematical model of EEG and EMG was proposed, the framework of the mathematical model was shown in Figure 3. The mathematical model consists of neural mass model (NMM), neuromuscular junction model, EMG generation model, decoding model, and musculoskeletal biomechanical model. In addition to its ability to systematically simulate EEG and EMG signals, the model can also determine the joint torque and movement intention.

In this paper, *P* (*t*) was defined as the input of movement intention. The *P* (*t*) will transmit to the motor cortex to generate EEG signals including RP response and the EEG signals simulated by NMM, the movement intention was recognized by the decoding model. Meanwhile, it also transmits to skeletal muscle to generate EMG through the nervous system and neuromuscular junction, the EMG signals are simulated by the neuromuscular junction model and the EMG generation model. Then, the joint torque caused by the joint's multiple muscles is decoded by the musculoskeletal biomechanical model. In this work, the nervous system is equivalent to a delay system, it only has a function of signal transmission and ignores the signal conversion function of the nervous system.

Overall, both EEG and EMG responses are triggered by the same movement intention *P* (*t*) during lower limb movement. It provides theoretical guidance for fusion of EEG and EMG.

#### Mathematical Model of EEG

Due to the complexity of EEG signals, modeling brain activity for the cortex can provide a theory of interpretation for how information generates and is transmitted in the motor cortex for different brain tasks (Rui et al., 2018a). Various mathematical models of brain activities could be subdivided into two major classes: “detailed model” and “neural mass model.” NMM is the dynamic of neural populations at a macroscopic level. To better understand the mechanism of EEG responses, a schematic diagram of NMM was shown in Figure 3. The RP responses were included in the proposed model construction in our previous work (Hanzhe et al., 2019a).

The stimulation of EEG responses can be obtained using coupled NMM based on the Jansen model (Jansen and Rit, 1995). The basic component of this model is the “cortical column” function (Zavaglia et al., 2006). In NMM, there are four neural groups: pyramidal cells, excitatory interneurons, slow inhibitory interneurons, and fast inhibitory interneurons. This model can simulate the EEG signals of the motor cortex in response to limb movement. The impulse responses of excitatory [*h*_*e*_ (*t*)] and inhibitory [*h*_*i*_ (*t*)] cells are as follows:

(6) $$\begin{array}{l} h_{e}\left(t\right)=\{\begin{array}{c} Aat{\text{e}}^{-at}\;t>0\\ 0\;t\leq 0 \end{array}\\ h_{i}\left(t\right)=\{\begin{array}{c} Bbt{\text{e}}^{-bt}\;t>0\\ 0\;t\leq 0 \end{array} \end{array}$$

Where *A, B* and *a, b* represent the average gain and time constant of excitatory and inhibitory cells, respectively. *h*_*e*_ = 3.25 mV, *h*_*i*_ = 22 mV.

The average postsynaptic membrane potential are the EEG signals that can be collected and observed. This conversion function *z* (*t*) is defined as follows:

(7) $$\begin{array}{l} z\left(t\right)=\frac{2e_{0}}{1+{\text{e}}^{r\left(s_{0}-v\left(t\right)\right)}} \end{array}$$

Where *e*_0_ is the maximum firing rate of neural population, *r* denotes the steepness of *z* (*t*), *v*_0_ is the potential synapses relative to *e*_0_, and *v* represents the input average postsynaptic membrane potential, *s*_0_ = 6 mV, *e*_0_ = 2.5 s^−1^, *r* = 0.56 mV^−1^.

The second-order differential equations were employed to combine *h*_*e*_ (*t*) and *h*_*i*_ (*t*). Differential equation groups used to calculate are as follows:

Pyramidal neurons:

(8) $$\begin{array}{l} \{\begin{array}{l} \frac{dy_{0}^{\;}\left(t\right)}{dt}=y_{5}^{\;}\left(t\right)\\ \frac{dy_{5}^{\;}\left(t\right)}{dt}=Aa_{1}^{\;}z_{0}^{\;}\left(t\right)-2a_{1}^{\;}y_{5}^{\;}\left(t\right)-{a_{1}}^{2}y_{0}^{\;}\left(t\right)\\ v_{0}\left(t\right)=C_{2}y_{1}\left(t\right)-C_{4}y_{2}\left(t\right)-C_{7}y_{3}\left(t\right) \end{array} \end{array}$$

Excitatory interneurons:

(9) $$\begin{array}{l} \{\begin{array}{l} \frac{dy_{1}^{\;}\left(t\right)}{dt}=y_{6}^{\;}\left(t\right)\\ \frac{dy_{6}^{\;}\left(t\right)}{dt}=Aa_{1}^{\;}\left(z_{1}^{\;}\left(t\right)+\frac{P\left(t\right)}{C_{2}}\right)-2a_{1}^{\;}y_{6}^{\;}\left(t\right)-{a_{1}}^{2}y_{1}^{\;}\left(t\right)\\ v_{1}\left(t\right)=C_{1}y_{0}\left(t\right) \end{array} \end{array}$$

Slow inhibitory interneurons:

(10) $$\begin{array}{l} \{\begin{array}{l} \frac{dy_{2}^{\;}\left(t\right)}{dt}=y_{7}^{\;}\left(t\right)\\ \frac{dy_{7}^{\;}\left(t\right)}{dt}=Bb_{1}^{\;}z_{2}^{\;}\left(t\right)-2b_{1}^{\;}y_{7}^{\;}\left(t\right)-{b_{1}}^{2}y_{2}^{\;}\left(t\right)\\ v_{2}\left(t\right)=C_{3}y_{0}\left(t\right) \end{array} \end{array}$$

Fast inhibitory interneurons:

(11) $$\begin{array}{l} \{\begin{array}{l} \frac{dy_{3}^{\;}\left(t\right)}{dt}=y_{8}^{\;}\left(t\right)\\ \frac{dy_{8}^{\;}\left(t\right)}{dt}=Gg_{1}^{\;}z_{3}^{\;}\left(t\right)-2g_{1}^{\;}y_{8}^{\;}\left(t\right)-{b_{1}}^{2}y_{3}^{\;}\left(t\right)\\ v_{1}\left(t\right)=C_{5}y_{0}\left(t\right)-C_{6}y_{2}\left(t\right) \end{array} \end{array}$$

Where, the *v*_*i*_ is the average membrane potentials for the four groups (*i* = 0, 1, 2, 3). The *z*_*i*_ (*i* = 0, 1, 2, 3) is the input for the sigmoid function which was fired by the neurons. *A, B*, and *G* are the average gain for the excitatory, slow inhibitory, and fast inhibitory synapses, respectively; *a*_1_, *b*_1_, and *g*_1_ are the time constant of the excitatory, slow inhibitory, and fast inhibitory synapses, respectively. The *y*_*i*_ (*i* = 0, 1, 2, 3) are outputs of these equations, which represent the postsynaptic membrane potentials of excitatory, slow inhibitory, or fast inhibitory, respectively. *C*_*i*_ represents the connectivity constants of those neurons for interactions among neurons. The *P* (*t*) is the input of model from motor or other cortex, which is represented by a Gaussian noise with assigned mean value (*m*) and variance (σ^2^) for movement intention.

A single NMM can simulate a unimodal spectrum for EEG response, its position and bandwidth can be adjusted appropriately. However, this model cannot simulate the complexity of EEG in the motor cortical area. Therefore, the model of the cortex is composed by multiple single-models deployed in parallel. Commonly, the coupling of three single-models is sufficient to simulate the motor task of cortical activity. Then, these models will be indicated with the symbols *L, M*, and *H* to represent rhythms at low, medium, and high frequencies of EEG, respectively.

#### Neuromuscular Junction Model

The neuromuscular junction is the contact point between motor nerve and skeletal muscle. The information conversion is achieved through the release of acetylcholine (ACh), and acetylcholine receptors (AChR) will be activated by it (Webster, 2018). Activated AChR will activate related ion channels to generate action potential leading to muscle contraction. In the past, the use of animal models has elucidated its formation and function by the cellular electrophysiology and patch clamp techniques. So, the circuit model of neuromuscular junction was established, the model structure was shown in Figure 3. This model simulates the current change of four kinds of ion flow and the change of membrane potential, it is a two-node circuit with multiple parallel branches (Guan et al., 2013). According to Ohm's law of active circuits, the current of each branch is as follows.

(12) $$\begin{array}{l} I_{i}=\frac{V_{m}-E_{i}}{R_{i}} \end{array}$$

Where *V*_*m*_ is the membrane potential. *I*_*i*_, *E*_*i*_, and *R*_*i*_ is the ionic current, equilibrium potentials and resistance of sodium (Na) ions branch, potassium (K) ions branch, chloride (Cl) ions branch and calcium (Ca) ions branch, respectively.

According to the constant field hypothesis proposed by Goldman, Hodgkin, and Katz, the algebraic sum of the branch current of each branch is zero at resting while ignoring the effect of the pump current. The resting potential of the membrane remains constant (Hodgkin and Huxley, 1952).

(13) $$\begin{array}{l} I_{K}+I_{Na}+I_{Cl}+I_{Ca}=0 \end{array}$$

Membrane potential (*V*_*m*_) can be obtained by combining Equations (12) and (13).

(14) $$\begin{array}{l} V_{m}\;=\;\frac{E_{k}g_{k}+E_{Na}g_{Na}+E_{Ci}g_{Ci}+E_{Ca}g_{Ca}}{g_{k}+g_{Na}+g_{Ci}+g_{Ca}},\;g_{i}\;=\;\frac{1}{R_{i}} \end{array}$$

Where *g*_*i*_ is the conductance of each ions branch.

According to the Nernst formula, the calculation of ionic equilibrium potential is as follows.

(15) $$\begin{array}{l} E_{i}=\frac{RT}{zF}\ln\;\left(C_{O}/C_{I}\right) \end{array}$$

Where *F* is the Faraday constant, *R* is the gas constant, *T* is the absolute temperature, and *z* is the changing number of electrons in the electrode reaction. *C*_*O*_ and *C*_*I*_ are the ion concentration outside and inside the membrane, respectively.

According to the physiological parameters of the human, the equilibrium potential of each ion branch can be obtained.

(16) $$\begin{array}{l} E_{K}=-91.2\text{mV, }E_{Na}=75.8\text{mV}\\ E_{Ca}=123.1\text{mV, }E_{Cl}=-123.8\text{mV} \end{array}$$

#### Mathematical Model of EMG

Human skeletal muscle is composed of a large number of muscle fibers (Wang et al., 2006; Jiangcheng et al., 2016). The end-plate potential is generated in the end-plate area of muscle fibers excited by the impulses of the neuromuscular junction, the action potential of a single fiber (*V*_*f*_) on muscle fiber membrane caused by end-plate potential. The action potential of the motor unit is generated by the action potential of all the muscle fibers in the motor unit, and the action potential sequence of the motor unit (*V*_*mu*_) is formed continuously in the time domain. Finally, the action potential sequences of different motor units are superimposed on the skin to form EMG signals (*V*_*s*_). According to the physiological process of EMG and its physiological basic structure, the EMG generation model was established, as shown in Figure 3. The simplified equation of the model is as follows.

(17) $$\begin{array}{l} V_{f}\left(x,y,z\right)=-\frac{\sigma_{i}}{4\pi \sigma_{i}}\int ds\int_{-\infty }^{\infty }\frac{\partial e_{i}\left(z\right)}{\partial z}\frac{\partial \left(1/r\right)}{\partial z}dz,\\ e_{i}=96{\left(vt\right)}^{3}e^{-vt}-90\\ V_{mu}=\overset{i=0}{\underset{N_{f}}{\sum }}V_{fi}\\ V_{s}=\left[\begin{array}{l} V_{\text{mut},1}\left(t_{\text{st},1},f_{\text{r},1},V_{\text{mu},1}\right)\\ V_{\text{mut},2}\left(t_{\text{st},2},f_{\text{r},2},V_{\text{mu},2}\right)\\ -\\ -\\ -\\ V_{\text{mut},j}\left(t_{\text{st},j},f_{\text{r},j},V_{\text{mu},j}\right)\\ -\\ -\\ -\\ V_{\text{mut},N}\left(t_{\text{st},N},f_{\text{r},N},V_{\text{mu},N}\right) \end{array}\right] \end{array}$$

Where *e*_*i*_ (*z*) is the intracellular action potential of a single fiber along the z direction; *s* is the cross-sectional area of muscle fiber; σ_*i*_ is intracellular conductivity; σ_*m*_ is muscle conductivity; σ_*x*_ is muscle radial conductivity; σ_*z*_ is fiber direction conductivity; *v* is the conduction velocity of action potential; *r* is distance between fiber's cross section and observation point. *T*_st,*i*_ is the burst time of action potential.

Equation (17) established the EMG signals model of the observation point. It can be seen that EMG signals are determined by two factors: the recruiting number of muscle fiber in motor units and firing rate of motor unit action potential.

#### Musculoskeletal Biomechanical Model and Decoding Model

The musculoskeletal model can obtain the joint torque from the original surface EMG signals (Jiangcheng et al., 2017). This model includes the following three sub models, which is muscle bioelectrical activation model, muscle contraction mechanical model, and muscular function model in the lower limb motor system. The first one uses EMG to characterize the degree of muscle activation *a* (*t*). The second model converts activation *a* (*t*) to output muscle force *F* (*t*). The third model converts muscle force *F* (*t*) to joint torque output *T*_*J*_. Those three models are formed according to the mechanical relationship. The output joint torque can be used in the interactive control of the robot.

(18) $$\begin{array}{l} \{\begin{array}{l} a\left(t\right)=\frac{A^{au\left(t\right)}-1}{e^{A}-1}\\ F_{\;}=F_{CE}+F_{PE}=\left(f_{l}\left(l_{m}\right)\cdot f_{v}\left(v_{m}\right)\cdot a\left(t\right)+f_{p}\left(l_{m}\right)\right)\cdot F_{0}\cdot \cos\varphi \\ T_{J}=\overset{n}{\underset{i}{\sum }}r_{i}\times F_{i} \end{array} \end{array}$$

Where, *u* (*t*) is the sEMG signal, *A* is the non-linear shape conversion factor, *F*_*CE*_ and *F*_*PE*_ are the muscle-force of contractile element (CE) and parallel elastic element (PE) in Hill model. *f*_*l*_ (*l*_*m*_) is force-length equation, *f*_*v*_ (*v*_*m*_) is force-velocity equation, *f*_*p*_ (*l*_*m*_) is parallel elastic force-length equation, *F*_0_ is maximum muscle contraction force, ϕ is pinnation angle, *r*_*i*_ is the moment arm of the *i*th muscle, *F*_*i*_ is the muscle force of the *i*th muscle.

The decoding model for EEG signals is a classifier which is based on artificial neural network (ANN) and deep learning, it provides a good performance in classifying inconspicuous feature sets. This model is an important way for humans to translate EEG signals into commands, it is also the research focus. Many scholars are committed to the study of excellent methods to improve the performance of this model, many classification methods have been presented in the numerous published EEG-based BCI articles (Rashid et al., 2020).

### Simulation Analysis

#### Simulation Analysis of EEG

A reference value of the parameters in the coupled NMM model have been given to simulate different bands of EEG signals. These values are reported in Table 1.

**Table 1:** Common and different parameters setup of coupled model.

**Parameters**	**Low frequency (*i* = L)**	**Medium frequency (*i* = M)**	**High frequency (*i* = H)**	**Connectivity constants**
*A^*i*^*	2.7	4.5	6.7	*C*_1_ = 135
*B^*i*^*	3.2	4.5	5.7	*C*_2_ = *C*_7_ = 108
*G^*i*^*	20.8	35.7	66.2	*C*_3_ = *C*_4_ = 33.75
*a^*i*^*	40	85	140	*C*_5_ = 40.5
*b^*i*^*	20	30	38	*C*_6_ = 13.5
*g^*i*^*	300	350	790	*s*_0_ = 6
*m^*i*^*	60.1	−20.6	115.9	*e*_0_ = 2.5
(σ^2^)*^*i*^*	61	30.5	69.0	*r* = 0.56

The theoretical results were obtained from simulation. Under the assumed conditions, the parameters of the activation function *P* (*t*) were defined as movement intention. In order to compare the difference between the movement intention state and the rest state, the activation function was defined as changing with the simulation time.

**Figure 4:**
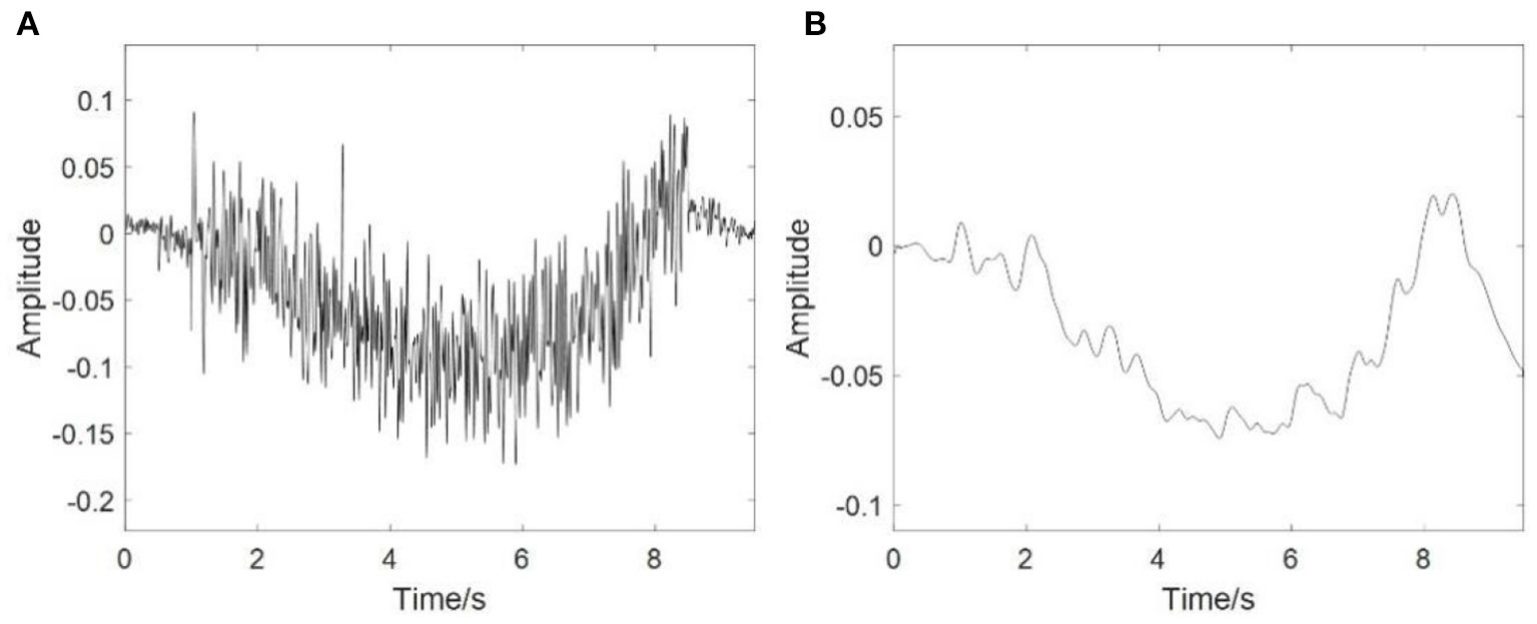
Simulation results of EEG. **(A)** The simulation results of the EEG signals, **(B)** The RP response from stimulation.

Figure 4A shows the simulation results of EEG signals, and Figure 4B shows an example of an RP stimulus. Clearly, the sharp drop and rapid recovery of low frequency potential is affected by the movement intention [*P* (*t*)]. The results also suggested that the coupled NMM could interpret the mechanism of the EEG including RP responses.

#### Simulation Analysis of Neuromuscular Junction Model

However, it is difficult to measure the resistance value of each ion branch in the neuromuscular junction model. The membrane potential change of the neuromuscular junction is caused by the difference in ion concentration between the inside and outside of the neuromuscular junction. Therefore, this characteristic is used to derive its voltage or current equation, Goldman, Hodgkin, and Katz deduced the GHK voltage equation based on Nernst-Plank equation. The membrane potential from GHK equation is as follows:

(19) $$\begin{array}{l} V_{m}=\frac{\left(RT/F\right)\text{ln}\left\{{\text{P}}_{\text{K}}{\left[{\text{K}}^{+}\right]}_{\text{O}}+{\text{P}}_{\text{Na}}{\left[\text{N}{\text{a}}^{+}\right]}_{\text{O}}+{\text{P}}_{\text{Cl}}{\left[\text{C}{\text{l}}^{-}\right]}_{\text{O}}+{\text{P}}_{\text{Ca}}{\left[\text{C}{\text{a}}^{2+}\right]}_{\text{O}}\right\}}{P_{K}{\left[{\text{K}}^{+}\right]}_{\text{I}}+P_{Na}{\left[\text{N}{\text{a}}^{+}\right]}_{\text{I}}+P_{Cl}{\left[\text{C}{\text{l}}^{-}\right]}_{\text{I}}+P_{Ca}{\left[\text{C}{\text{a}}^{2+}\right]}_{\text{I}}} \end{array}$$

Where, *F* is the Faraday constant, *R* is the gas constant, *T* is the absolute temperature, *P*_*i*_ is the permeability constant of each ions, [*i*]_*O*_, [*i*]_*I*_ are the ion concentrations outside and inside the membrane, respectively.

**Figure 5:**
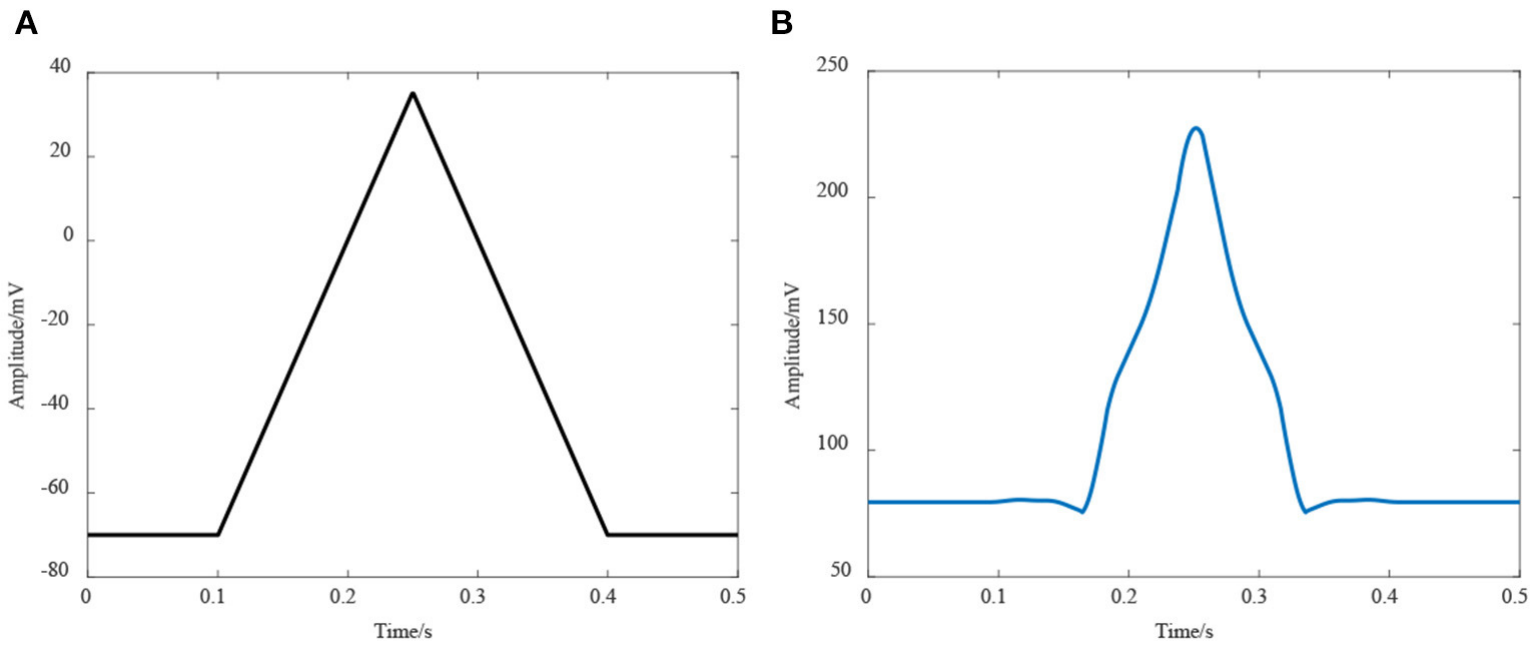
Simulation of the neuromuscular junction model. **(A)** The activation function of the model; **(B)** Simulation result of this model.

It can be seen from the formula that the membrane potential depends on the transient ion concentration, but it is different to measure the value. At present, it is usually measured by patch clamp recording technique technology, and the *I*-*V* curve of the ion branch was obtained (Guan et al., 2013). Although this is a voltage-gated current curve, it can reflect the characteristics of the ion branch. In this work, the resistance value of each ion branch is calculated according to its *I*-*V* curve, and the activation function of this model was defined as a trigonometric function similar to the action potential received by the neuromuscular junction, whose shape is shown in Figure 5A.

The simulation result is shown in Figure 5B, it can be seen that the membrane potential of the neuromuscular junction had a considerable consistency with the real end plate potential. Furthermore, a significant delay in response time was found in the simulation result. Although this change is due to the charge and discharge of membrane capacitance in this model, it still reflects a time required for neuromuscular junction to convert the information. Even though the accurate cost time of conversion cannot be obtained from this model, a qualitative analysis has a certain value. This result suggested that the control signal from the brain and nervous system through neuromuscular junction received by the muscle fiber is further delayed.

#### Simulation Analysis of EMG

**Figure 6:**
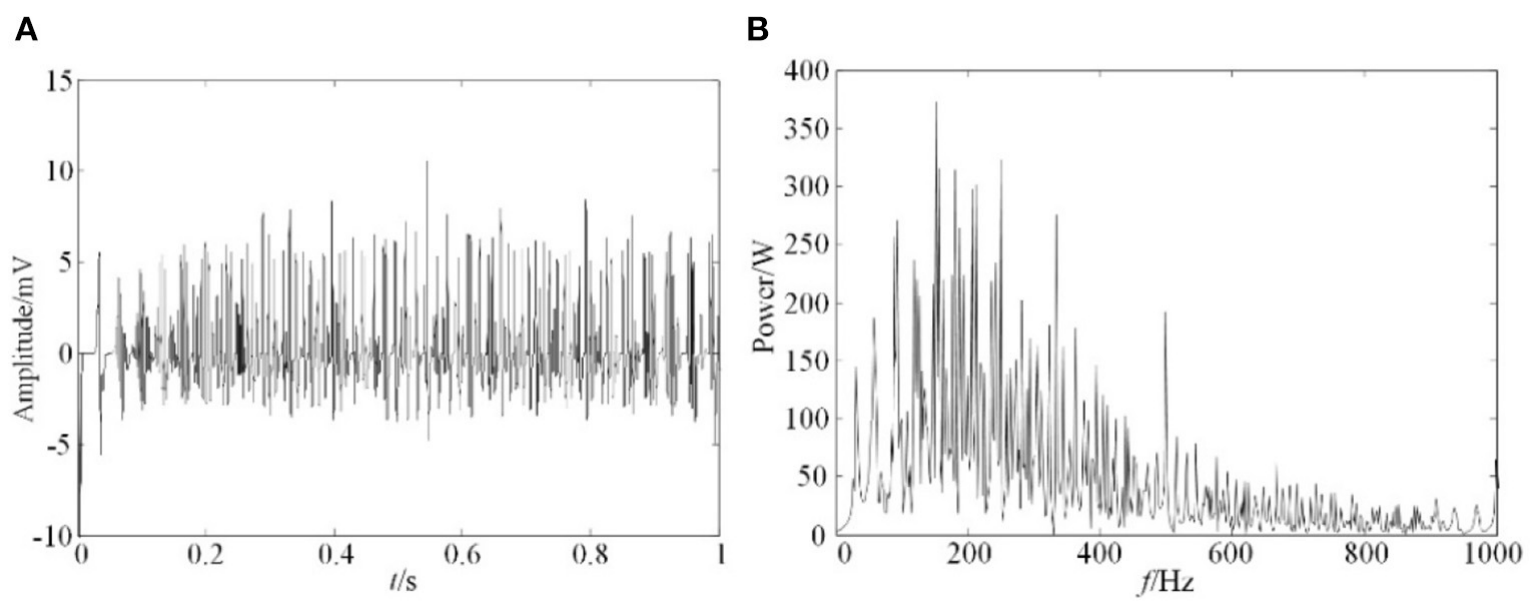
Simulation result of EMG. **(A)** EMG simulation signals, **(B)** Spectrum analysis of EMG simulation signals.

According to the physiological knowledge and experience, the relevant parameters of the model were given. The cross-sectional area of muscle fiber *s* was 2,300 μm^2^, the total number of muscle fibers *N*_*f*_ can be calculated as 127,235 by the distribution density of muscle fibers, when muscle diameter was set as 45 mm. The firing frequency of the action potential *f*_*r*__,*i*_ follows the Poisson distribution between 8 and 50 Hz, with an average value of 12 Hz. The sampling frequency was set as 2 KHz. The simulation results and its spectrum analysis from this model was obtained, as in Figure 6.

The results shown in Figure 6 indicated that the amplitude of EMG signals was between 0 and 10 mv, and its frequency distribution range is mainly between 0 and 400 Hz. This result was consistent with measured sEMG signals, which further proved the correctness of this simulation model.

Each model has well-simulated the EEG signals, conversion process of neural signals, and EMG signals. These simulation results explain the characteristics of EEG and EMG signals very well; they also explained the internal relationship between EEG and EMG signals at the same time. In the simulation of this work, the musculoskeletal biomechanical model was ignored, because this model is a signal decoding function. In general, the simulation of the model also proves the difference in the response time between EEG and EMG. The results indicated that the response of EEG signals is earlier than EMG signals, which proves the proposed theory in this paper.

#### Simulation Analysis for Homology Characteristic of EEG and EMG

**Figure 7:**
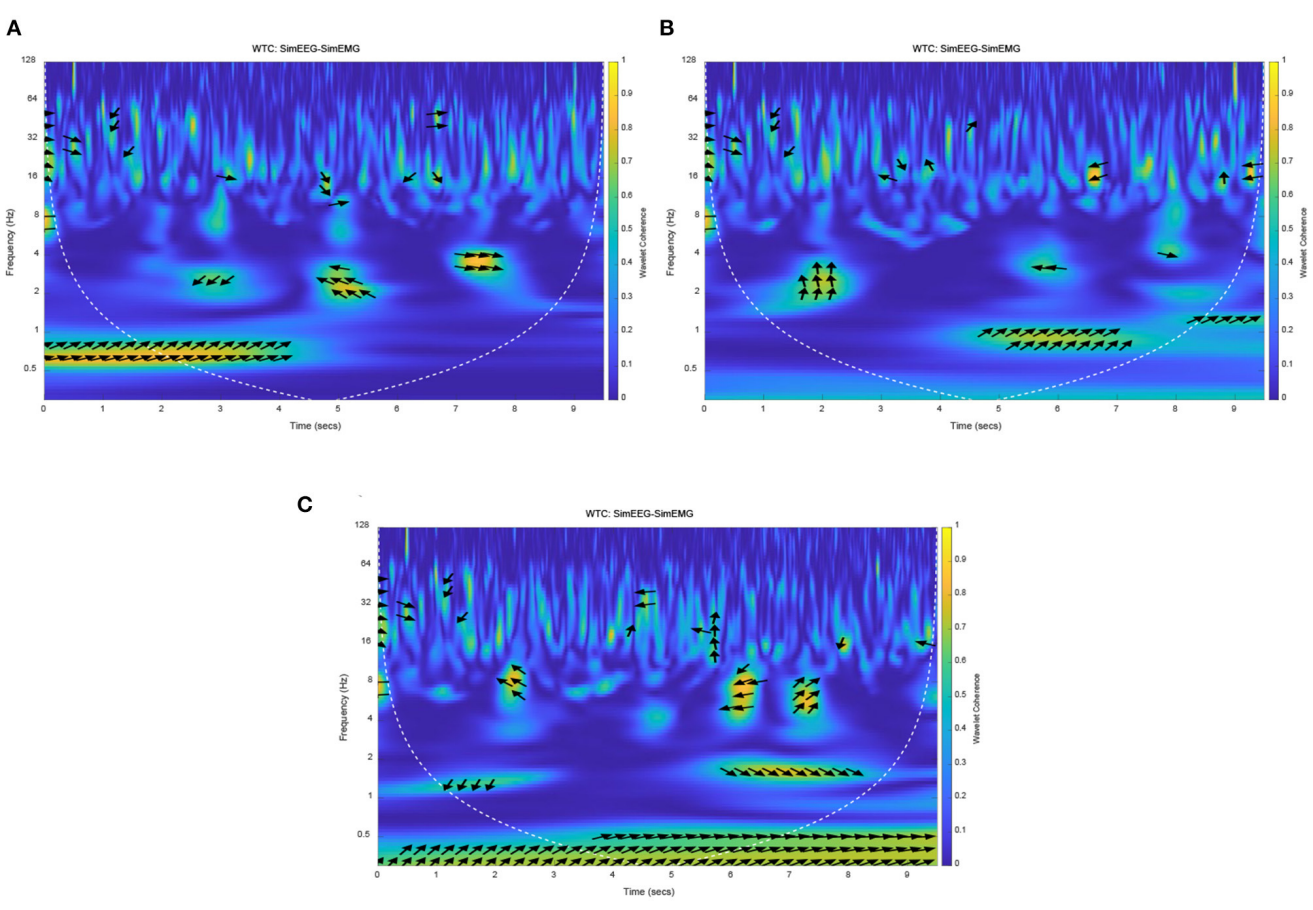
The Wavelet coherence of the simulation of EEG and EMG. **(A)** EEG-EMG coherence at early EMG response, **(B)** EEG-EMG coherence at middle EMG response, **(C)** EEG-EMG coherence at late EMG response.

Because the connection characteristics of each functional model in this mathematical model are imperfect, the delay time caused by transfer and conversion is especially unclear. Therefore, different response times of EMG were set to observe the coherence of EEG and EMG. Due to the properties of simulation and real EEG and EMG signals, the simulation EMG signals were normalized, and EMG signals of resting state were added before and after task state. The EMG signal response time (movement onset) was set to three cases, which was set at the early, middle and late stages of the EEG signal response based on an exhaustive method. So, the EMG response onset time was set at 2 5 and 7 s, respectively, and the duration was 3 s. The wavelet coherence of simulation EEG and EMG signals were obtained, as shown in Figure 7.

The yellow areas in Figure 7 represented the strong simulation of EEG-EMG coherence. It can be seen that the frequency band and time of strong coherence in those figures were different with different EMG response times. Especially for the early and late EMG response (seen in Figures 7A,B, respectively), the time of strong coherence was not consistent with the response time of EMG signals. But for Figure 7B with the EMG response onset at the middle of the EEG signal response, there was a strong coherence at 1 Hz after the EMG response onset (from 5 s to 7 s). This result accords with theory and practice in terms of response time and frequency band, this result indicated that the correct setting of EMG response onset time can obtain a correct simulation result, and response of EEG signals is earlier than that of EMG signals. It also can be seen that the simulation phase of the EEG signals was leading the EMG phase at 1 Hz in Figure 7. The simulation results will be verified with the experimental results, and then used to analyze and verify the time difference of EEG and EMG response. The coherence and phase of simulation EEG and EMG signals proved the homology and different response times of simulation EEG and EMG signals, which was consistent with the theoretical analysis and proposed hypothesis.

## Experimental Verification for Homology Characteristic of EEG and EMG

### Experimental System Overview

In this work, the EEG and EMG signals of lower limb voluntary movement intention acquisition system comprised an EEG and EMG acquisition device and signals processing equipment. The EEG and EMG data were acquired using NeuroScan NuAmps. A PC with Intel (R) Core (TM) i5-5600 CPU was used to process EEG and EMG signals.

**Figure 8:**
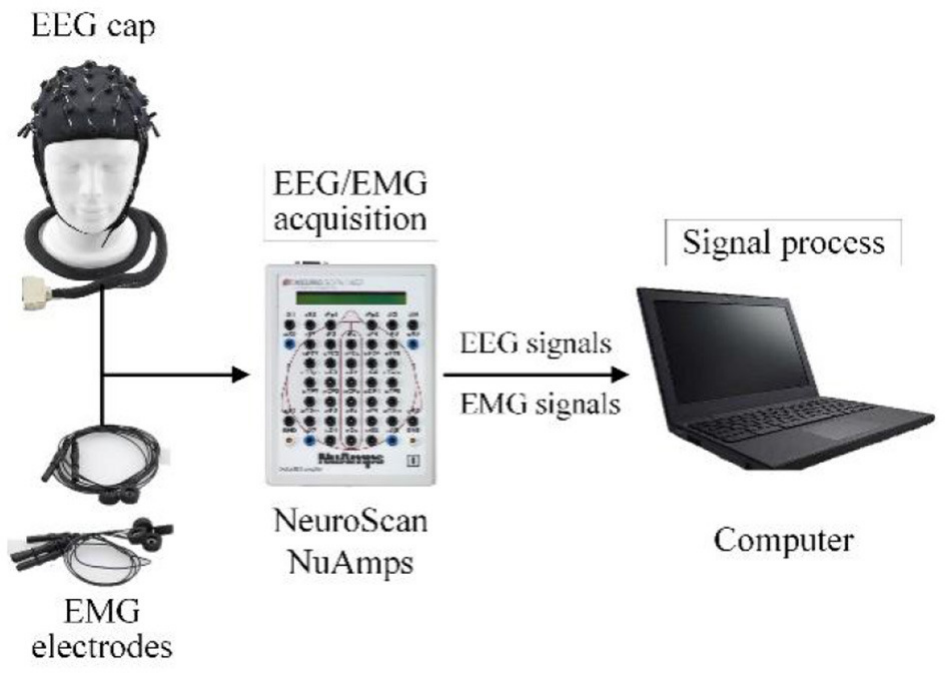
The schematic description of the EEG and EMG acquisition system.

A diagram of the EEG and EMG acquisition system is shown in Figure 8. When the system was working, the subject performed the voluntary movement. At the same time NeuroScan-NuAmps recorded the EEG from the subject's motor cortex and EMG data were recorded with same equipment from subject's tibialis anterior. All the recorded data were transferred to the PC by USB. The computer processed the EEG and EMG data with special algorithms.

### Subjects and EEG and EMG Data Recording

Six healthy subjects participated in this experiment (five males and one female, aged 24–28, mean 23.2 years). None of them has limb dysfunction or any known cognitive deficits. After explaining the nature and possible consequences of the experiment, all the subjects signed the informed consent. The Institutional Review Board of Xi'an Jiaotong University approved the proposed experiments and all experiments were conducted in accordance with the declaration of Helsinki.

EEG and EMG data were acquired using NeuroScan-NuAmps with sampling rate at 1,000 Hz. The system had 36 EEG channels. The channel distribution was in accordance with the international 10/20 system. Channels FCz, FC1, FC2, Cz, C1, C2, CP1, and CP2 were selected. Channels AFz and CPz were used as references. The raw EEG signals were first filtered by a 0.5–45 Hz band pass filter. 2 EMG channels from tibialis anterior were selected to offer EMG data detection. The raw EMG data was filtered by a 20–200 Hz band pass filter and a 50 Hz notch filter.

### Experimental Procedure

**Figure 9:**
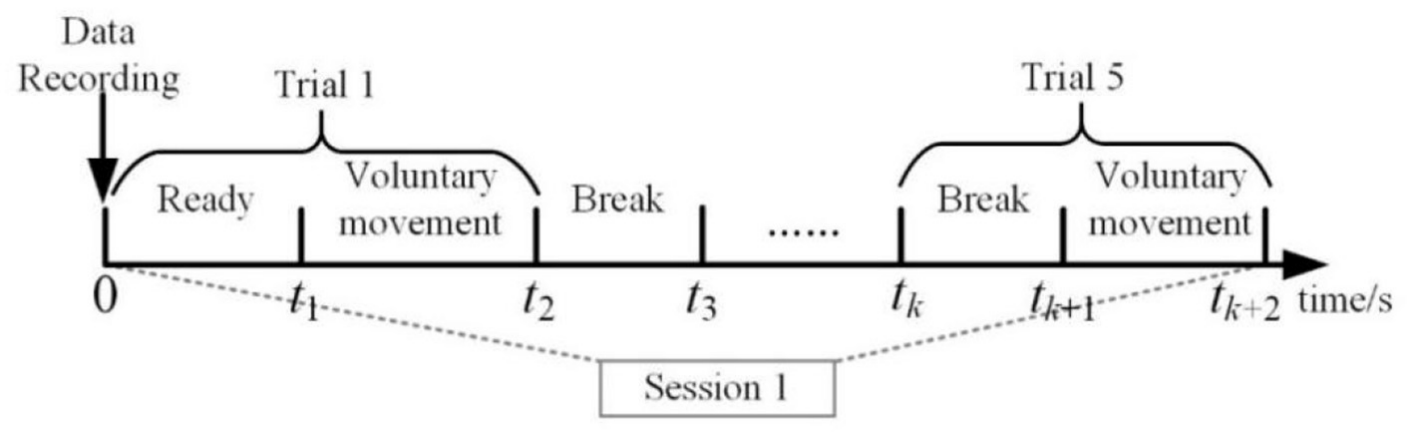
Overview of the time series of one session.

This experiment was conducted to verify the interconnection of EEG and EMG. Each subject completed experiments on several separate days. The subjects were instructed to be in a standing position. In this experiment, the subjects should keep the resting state to avoid unnecessary movement. The subjects were asked to perform the lower limb voluntary movement tasks. There were two parts of voluntary movement tasks, including the left and right leg voluntary movement. Each part consisted of 15 sessions. In each session, all subjects repeated the same lower limb voluntary movement five times. The experiment was conducted in a sequence of intervals between breaks and voluntary movements. Each break time exceeds 10 s, the rest time between each two sessions was decided by the subjects themselves. Figure 9 is the time series for this experiment.

### Coherence Analyses for Homology Characteristics of EEG and EMG

**Figure 10:**
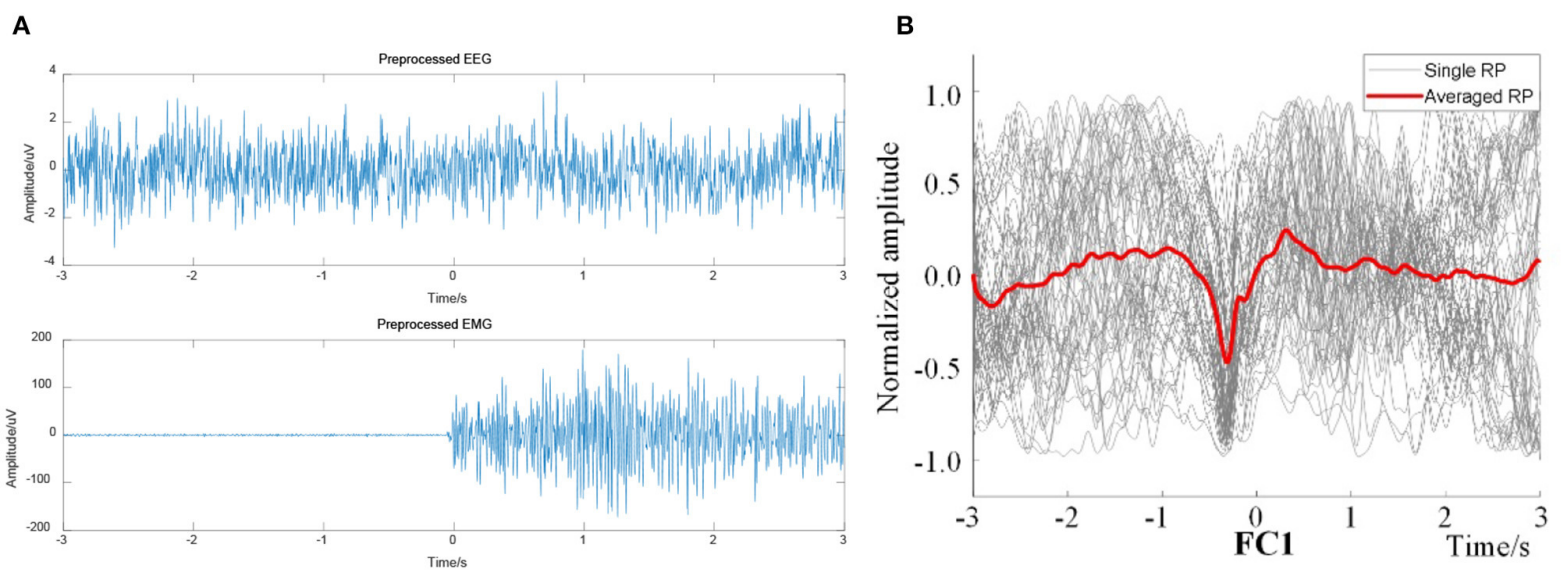
Preprocessed EEG and EMG signals from right leg movement. **(A)** Preprocessed EEG (representative channel FC1) and EMG signals, **(B)** Single and averaged RP of FC1.

The preprocessed EEG signals and the EMG signals were computed to obtain the EEG–EMG coherence curves under the two actions of right leg and left leg voluntary movement. The onset trigger of lower limb voluntary movement was taken as the data center. So, the EEG and EMG data were segmented from continuous data streams. Under this definition, EEG data were segmented to complete the event alignment. The preprocessed EEG and EMG was shown in Figure 10. At the same time, the results were computed after ERP analysis of EEG signals (as shown in Figure 10B), it validated that there had been a significant RP response in the motor cortex (representative channel FC1) during right leg voluntary movement. There are potential changes in accordance with the characteristics of readiness potential at the onset of lower limb voluntary movement. This analysis validated that the experimental results were consistent with the simulation results, which proves that there is a RP response in the EEG of the motor cortex before the onset of lower limb voluntary movement.

**Figure 11:**
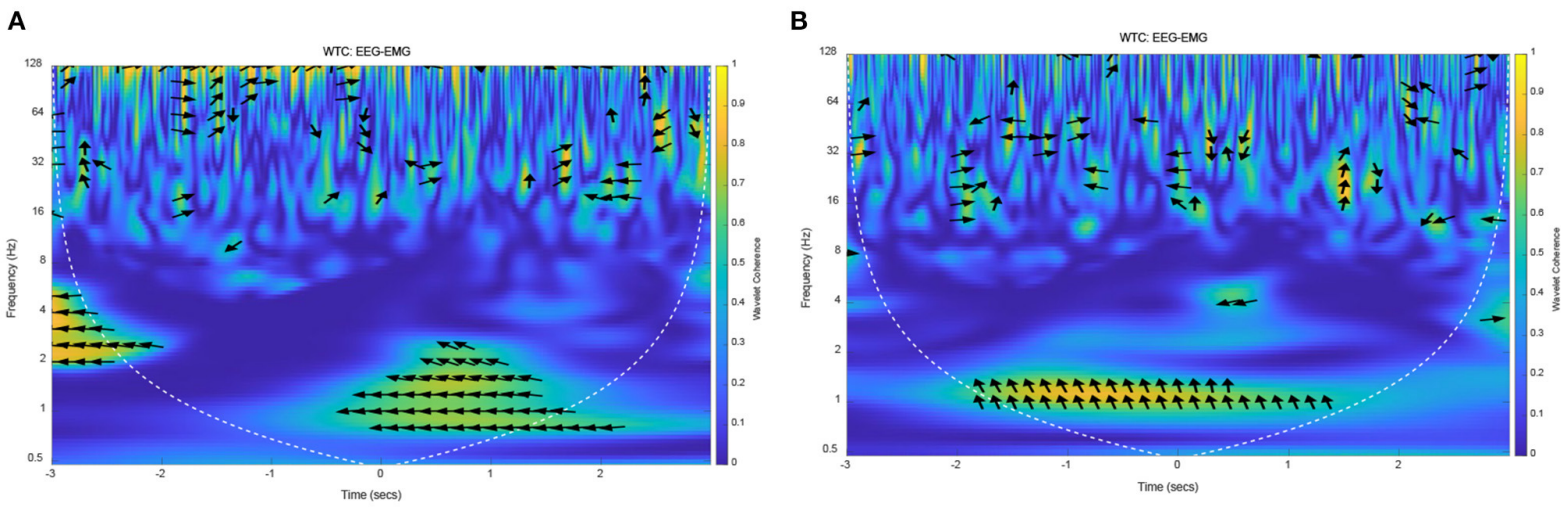
The wavelet coherence of EEG and EMG from representative subject (S1). **(A)** EEG (FC1) and EMG signals from right leg movement, **(B)** EEG (C4) and EMG signals from left leg movement.

On the basis of the above methods and data, EEG–EMG wavelet coherence can be obtained by selecting the tibialis anterior that dominate the lower limb voluntary movements. The wavelet coherence of EEG and EMG signals from one representative subject (S1) were obtained, as shown in Figure 11.

The yellow areas in this Figure 11 represented the strong EEG-EMG coherence at 1 Hz, in which the values are substantially increased relative to other frequencies. In Figure 11A, there was a strong coherence in the low frequency after right leg voluntary movement (from 0 to 2 s). At the same time, there also was a strong correlation at 1 Hz at the left leg voluntary movement (as shown in Figure 11B). This result suggested that the EEG and EMG signals are homologous, they were both the expression of lower limb voluntary movement due to the strong correlation at the onset of movement. It also can be seen that the phase of the EEG was leading the EMG phase at 1 Hz, while there was no obvious regularity in the distribution of coherence and phase difference in other frequency bands. Accordingly, this result was consistent with the above theoretical analysis and simulation results, which proved that the response of EEG signals was leading the EMG signals, and had a consistency and universality in the lower limb voluntary movement. Meanwhile, the experimental results also proved the correctness of the simulation results. There is a strong correlation at 1 Hz because there is a low-frequency MRCP in EEG signals response, and the EMG signals respond at this time. Although the low-frequency part of the EMG was filtered in the preprocessing process, the envelope of its amplitude shows a low-frequency characteristic, thus it shows a strong coherence at this frequency and at the movement onset.

**Figure 12:**
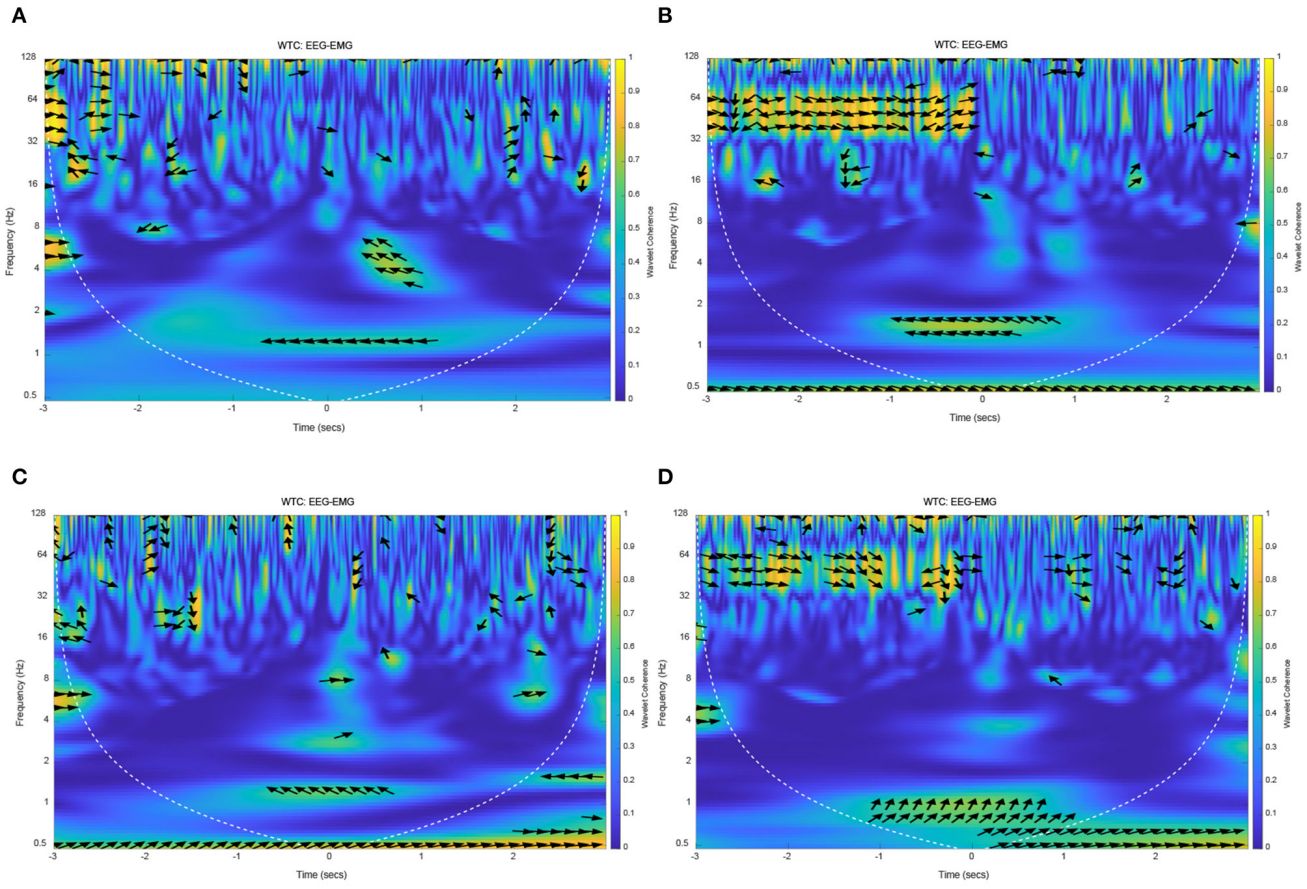
The wavelet coherence of EEG and EMG from other subjects. **(A)** Wavelet coherence of EEG (C1) and EMG signals from S2, **(B)** Wavelet coherence of EEG (C1) and EMG signals from S3, **(C)** Wavelet coherence of EEG (CP1) and EMG signals from S4, **(D)** Wavelet coherence of EEG (FC2) and EMG signals from S5.

The results of EEG and EMG coherence analysis of other subjects were shown in Figure 12. These results showed that there was a strong coherence between EEG and EMG at about 1 Hz during lower limb voluntary movement onset. The arrows pointing indicate the phase difference between the EEG and EMG, the distribution and direction of these arrows are basically consistent with the above results. It suggested that the results and conclusions of the above analysis were universal.

## Discussion

In this work, the mathematical model of EEG and EMG was presented and used to explain the homology characteristic between EEG and EMG signals for lower limb voluntary movement.

### The Significance of the Mathematical Model

With regards to the EEG model, it verified that the motor cortices are responsible for lower limb voluntary movement. As is known to all, the processing of lower limb voluntary movement in the cortical regions of brain is complicated, requiring different information sources and presentation. However, there is no doubt that the motor cortex provides critical contributions to lower limb voluntary movement processing. The EEG model can simulate the EEG signals in the motor cortex when a human generates a lower limb voluntary movement intention. Compared with our previous modeling (Hanzhe et al., 2019a), the coupling model can better explain the mechanism of the RP responses. Despite its relative simplicity, this mathematic model can explain the response mechanism of RP responses to a lower limb voluntary movement intention.

The neuromuscular junction is an important bridge between nerves and muscles, which realizes the transformation from electrical signals transmission to chemical transmission and then to electrical signals transmission. Under the stimulation of nerve action potential, the release and flow of chemical transmitters and ions in neuromuscular junctions causes changes of its current and membrane potential. Based on its working mechanism, the neuromuscular junction model is proposed, which can simulate the changes of the current and endplate potential.

The EMG generation mode received the control signal from the neuromuscular junction, which activated the skeletal muscle of the lower limb to generate an EMG signal. The simulation result showed the time and frequency characteristics of signals, and it can guide the subsequent signal processing and feature extraction.

Importantly, most of the existing EEG and EMG-based control technologies have some shortcomings, because they are based on empirical evidence or experimental results. Thus, a mathematic modeling can further understand the physiological mechanisms for the responses of EEG and EMG behavior (Rui et al., 2018a). Physiological analyses and model simulations of neural pathways of EEG and EMG show that there are significant differences in the generation and transduction pathway of EEG and EMG, but the EEG and EMG signals were homologous. The EEG signal response times are earlier than EMG signals. The results presented in this work were investigated for the feasibility of EEG-based pre-perception and fusion perception of EEG and EMG for human movement.

### The Homology of EEG and EMG Signals

With regards to the homology of EEG and EMG signals, the simulation and experimental results show a high consistency and the strong coherence appears at about 1 Hz during lower limb voluntary movement onset. Compared with the previous research results, the frequency band of the strong coherence of EEG and EMG is significantly different, in which the significant EEG-EMG coherence was observed broadly within 10–30 Hz (Mima et al., 2000a). Since the beta band plays an important role in information processing, it sees the cortico-muscular information flow in this frequency range. The reason for this difference is that this work focused on the readiness potential at low-frequency. Although the there was a divergence, the same conclusion is that the motor control command is from the cortex to the muscle. This conclusion agrees with our previous hypothesis that the EEG and EMG signals are homologous. What is more, the cortical-muscular coherence concerns the time lag between EEG and EMG, the results show that the EEG precedes and drives the synchronization of motor unit activity (Mima et al., 2000b). The phase of EEG-EMG coherence analysis means that the cortical oscillation leads the EMG rhythm, this result proves our previous hypothesis that the EEG response is earlier than EMG response.

Although the work has proved the homology and different response times of EEG and EMG signals, there are still some concerns. The EEG data were acquired for motor cortex, but the EMG data were only collected from the tibialis anterior, if the position of the EMG acquisition is changed, the result may change. What is more, the individual difference is an important factor to the result. It can be seen that the results have good consistency from this work, but it is still clear that the differences of some results are caused by individual differences.

### The Limitations of the Study and Further Work

Despite the significance of this work, several limitations should be considered. One of the limitations is that the proposed model was simplified, especially in the part of signal transmission and conversion from brain to muscle. It is known that the transmission and conversion of human nerve signals is very complicated, it involves multiple human organs and neural pathways. The purpose of this paper is to explore the intrinsic relationship between EEG and EMG signals of lower limb voluntary movement, and to provide theoretical support for the decoding and fusion decoding of EEG and EMG. Therefore, the functions of the low-level central nervous system and peripheral nervous system were simplified into a time-delay system with only nerve signal transmission functions. This work was based on this hypothesis to simulate and analyze EMG signals. From the overall perspective of the proposed model, when producing lower limb voluntary movement intention, it will stimulate the relevant regions of the brain to generate responses and EEG signals; at the same time, it will control the muscles through the transmission and conversion of the nervous system and neuromuscular junctions and generate EMG signals. Besides, the closeness between simulated EEG/EMG and real EEG/EMG signals is a lack of quantitative evaluation. In this work, it was the qualitative evaluation based on observation. For the EEG signals, it focused on the RP. While the simulated EEG and real EEG had the same trend of RP, it is considered that they have a good similarity. For the EMG signal, it mainly evaluated the closeness based on the spectrum distribution of EMG signals. In the following work, the non-linear correlation and time-frequency parameters of EEG/EMG signals will be employed for quantitative evaluation.

Another limitation of this study is that only six subjects participated in this experiment. Even though the number of subjects is not enough, it is sufficient to prove the correctness of the proposed viewpoint and highlight some significant experimental results. There is no doubt that the main motivation for this work is to provide a theoretical support for the fusion of EEG and EMG, which improve the movement intention recognition performance. Besides, there was a gender imbalance among subjects. Further study will involve more subjects testing the proposed hypothesis and result. Moreover, significant differences were not found between male and female subjects in this work. Thus, a large sample size and a balance between male and female subjects are desired to further evaluate the correctness of the proposed hypothesis and results. In future works, the specific time difference between EEG and EMG signals will be studied to take advantage of the two signals to improve the performance of human movement intention detection, so as to realize the interactive control applied in robot system.

## Conclusion

In this work, a numerical simulation and homology analysis of EEG and EMG from lower limb voluntary movement intention was conducted. The mathematical model of EEG and EMG from lower limb voluntary movement intention was proposed based on the electrophysiological analysis, which revealed the mechanism of EEG and EMG signals and the homology of those two signals. This model systematically reveals the generation and transmission mechanism of EEG and EMG signals. Especially, the internal relationship between EEG and EMG has a clear exposition, from which the difference in response time has been shown from the transmission pathway in this model. The simulation results of the neuromuscular junction model prove that the conversion of the neural signal takes a certain time. Meanwhile, the homology analysis results of the simulation signals show that the correct setting of the EMG response onset time can obtain a correct simulation result. In the experiment, this work synchronously collected the EEG and EMG signals of lower limb voluntary movement, and the experimental results are consistent with the hypothesis and simulation. Overall, both simulation and experimental results demonstrate the homology of EEG and EMG, and that the EEG signals response times are earlier than those of EMG signals. This work can provide a basis for the feasibility of EEG-based pre-perception and fusion perception of EEG and EMG in human movement intention detection.

## Data Availability Statement

The raw data supporting the conclusions of this article will be made available by the authors, without undue reservation.

## Ethics Statement

The studies involving human participants were reviewed and approved by Institutional Review Board of Xi'an Jiaotong University. The patients/participants provided their written informed consent to participate in this study.

## Author Contributions

XZ proposed the research idea and supervised the work. HL did the research and wrote the manuscript. ZL organized and carried out the experiments. GY revised the manuscript. All authors contributed to the article and approved the submitted version.

## Conflict of Interest

The authors declare that the research was conducted in the absence of any commercial or financial relationships that could be construed as a potential conflict of interest.
